# Screen Key Genes Associated with Distraction-Induced Osteogenesis of Stem Cells Using Bioinformatics Methods

**DOI:** 10.3390/ijms22126505

**Published:** 2021-06-17

**Authors:** Jishizhan Chen, Jia Hua, Wenhui Song

**Affiliations:** 1 UCL Centre for Biomaterials in Surgical Reconstruction and Regeneration, Division of Surgery & Interventional Science, University College London, London NW3 2PF, UK; jishizhan.chen.19@ucl.ac.uk; 2 UCL Institute of Orthopaedics and Musculoskeletal Science, Division of Surgery & Interventional Science, University College London, Stanmore, London HA7 4AP, UK; j.hua@ucl.ac.uk; 3 The Griffin Institute (Northwick Park Institute for Medical Research), Harrow, London HA1 3UJ, UK; 4 Faculty of Science and Technology, Middlesex University, London NW4 4BT, UK

**Keywords:** bioinformatics, distraction osteogenesis, gene expression, microarray, mesenchymal stem cells

## Abstract

**Background**: Applying mesenchymal stem cells (MSCs), together with the distraction osteogenesis (DO) process, displayed enhanced bone quality and shorter treatment periods. The DO guides the differentiation of MSCs by providing mechanical clues. However, the underlying key genes and pathways are largely unknown. The aim of this study was to screen and identify hub genes involved in distraction-induced osteogenesis of MSCs and potential molecular mechanisms. **Material and Methods:** The datasets were downloaded from the ArrayExpress database. Three samples of negative control and two samples subjected to 5% cyclic sinusoidal distraction at 0.25 Hz for 6 h were selected for screening differentially expressed genes (DEGs) and then analysed via bioinformatics methods. The Gene Ontology (GO) terms and Kyoto Encyclopaedia of Genes and Genomes (KEGG) pathway enrichment were investigated. The protein–protein interaction (PPI) network was visualised through the Cytoscape software. Gene set enrichment analysis (GSEA) was conducted to verify the enrichment of a self-defined osteogenic gene sets collection and identify osteogenic hub genes. **Results:** Three hub genes (*IL6*, *MMP2*, and *EP300*) that were highly associated with distraction-induced osteogenesis of MSCs were identified via the Venn diagram. These hub genes could provide a new understanding of distraction-induced osteogenic differentiation of MSCs and serve as potential gene targets for optimising DO via targeted therapies.

## 1. Introduction

Segmental long bone defects caused by high-energy trauma, traffic accident, and military activities remain a surgical challenge. There are more than 4.5 million bone reconstruction procedures worldwide, and bone defects lead to significant negative consequences or disability if not managed using appropriate approaches [1,2]. Distraction osteogenesis (DO) and the Ilizarov external ring fixator developed by G.A. Ilizarov have now been acknowledged in the orthopaedic world as one of the most important strategies for bone defect reconstruction [2,3]. DO procedures comprise three phases: the latency phase after the application of external fixation, the distraction phase implementing gradual and continuous distraction, and the consolidation phase for bone quality strengthening and bone remodelling [4]. However, DO relies on the recruitment, proliferation, and differentiation of mesenchymal stem cells (MSCs) at the target site to promote bone formation, which is a slow process [5]. This results in one of the main hurdles that patients must wear distraction devices throughout the long period of treatment and bear the risks of discomfort, psychological problems, and complications. Hence, there emerges an urgent need for shortening the distraction phases and accelerating DO.

Applying extraneous MSCs on bone regeneration has been widely investigated and shows promising potential [6,7]. Among multifarious sources of MSCs, bone marrow-derived MSCs (BMSCs) are recognised as the bona fide skeletal stem cells and the natural source of bone regeneration [8]. For the purpose of enhancing bone regeneration, a number of in vitro and in vivo studies analysed the combination of MSCs cultures and DO and made remarkable progress. The up-to-date data demonstrate that stem cell treatment during DO increases bone quality, volume, mineral density, trabecular thickness, and biomechanical strength [9,10,11]. MSCs have long been established as mechanosensitive cell types. In recent decades, researchers studied how MSCs transduced mechanical signals into biochemical signals leading to gene transcription. The osteogenic differentiation of MSCs is thought to occur in part through direct mechanotransduction of physical stimuli from the cellular microenvironment [12]. In vitro, the elasticity and topography of extracellular matrix (ECM) and external mechanical forces guide MSCs phenotype, proliferation, and differentiation. The cytoskeletal systems of MSCs sense mechanical stimuli mainly via focal adhesions and transduce into inner cellular compartments via actin filaments and microtubules [13]. Studies in the literature show that some genes were discovered to be regulated by DO. For example, interleukin 6 (*IL-6*), tumour necrosis factor-alpha (*TNF-α*), insulin-like growth factor 1 (*IGF-1*), bone morphogenetic protein 2 and 4 (*BMP-2, -4*), transforming growth factor-beta (*TGF-β*), vascular endothelial growth factor (*VEGF*) family, and other genes displayed varying expression patterns at different phases of DO [14]. Despite partially known the contribution of mechanical loading in osteogenesis, the underlying mechanism by which the cells sense and transduce into gene expression levels remains unclear. Most of the studies adopted the method of verifying the change of previously discovered bone-related genes when analysed the influences caused by DO; however, this method may not be efficient for identifying new key genes specifically mediated by DO. Still, there is very few studies that give insight to the gene expression patterns of MSCs’ sustained distraction without adding exogenous chemical molecules. Therefore, this study focuses on identifying key genes participating in the DO-induced osteogenesis of MSCs. The understanding of biomolecular mechanisms that mediate the response of MSCS to DO can give guidance to the development of more targeted strategies aimed at improving DO outcome, accelerating bone regeneration, and potentially shortening the treatment time.

ArrayExpress database is a comprehensive public repository archive that stores a variety of disease gene expression profile datasets from high-throughput functional genomics experiments [15]. Mining hub genes using bioinformatics methods provides new insight into the pathogenesis of complex diseases, whereas few studies have been conducted on gene expression profiling of DO-induced osteogenesis of MSCs. Additionally, to the best of our knowledge, there is no study that has launched an in-depth microarray analysis. Here, we first performed a series of analysis, including differentially expressed genes (DEGs) identification, Gene Ontology (GO) terms enrichment, Kyoto Encyclopaedia of Genes and Genomes (KEGG) pathway analysis, and protein–protein interaction (PPI) network analysis, to screen hub genes that respond to distraction on a general scale. Subsequently, gene set enrichment analysis (GSEA) was utilised for further identification. GSEA is a potent tool used for verifying the enrichment of specific osteogenesis-related gene sets in groups receiving different treatments. To accomplish this, we constructed a collection containing 19 osteogenesis-related gene sets. This collection was utilised in GSEA to screen out hub genes associated with osteogenesis further, and without relying on previous reports. This strategy is beneficial to discover genes that had previously been overlooked, and these findings may provide a new perspective for optimising the treatment of DO.

## 2. Results

### 2.1. Identification of DEGs

**Figure 1:**
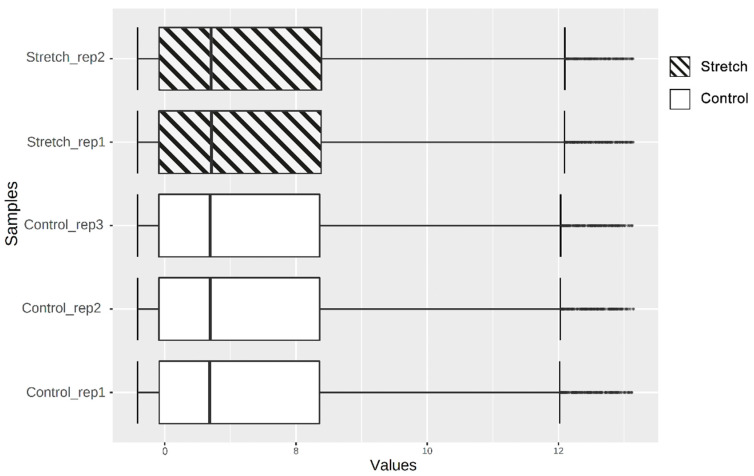
Boxplot of quantile normalised data. Vertical black lines in the boxes represent medians.

**Figure 2:**
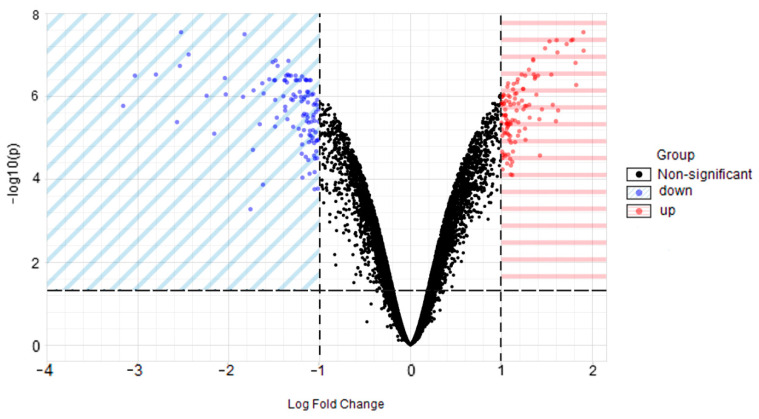
Volcano plot of all genes detected in the microarray. Each dot represents a gene. Dashed lines divide areas of down- and upregulated genes. The X-axis is log2-base fold change, and Y-axis is −log10-base adjusted *p*-value.

**Figure 3:**
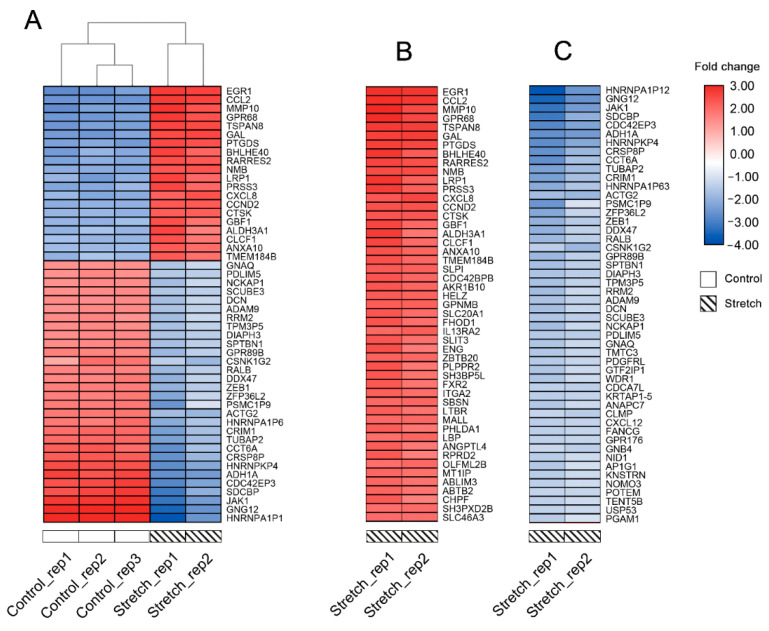
(**A**) Cluster heatmap demonstrates top 50 DEGs with the greatest absolute fold change, and hierarchical clustering analysis results according to groups. Each row represents a DEG, and each column represents a sample; (**B**) top 50 upregulated DEGs of distraction group; (**C**) top 50 downregulated DEGs of distraction group. The colour displays the fold change. Red indicates upregulation in gene expression, and blue indicates downregulation. The darker in colour, the greater fold change.

Figure 1 displays the gene expression data after quantile normalisation. A total of 220 DEGs were obtained, of which 108 (49.09%) were upregulated genes, and 112 (50.91 %) were downregulated genes in distraction-treated, human-bone-marrow-derived MSCs (hBMSCs), compared to control groups. Volcano plot (Figure 2) demonstrates the differential expression status of all detected genes while highlighting DEGs beyond the set cut-off criterion. The cluster heatmap of DGEs is displayed in Figure 3. Significant differences in DEG expression can be observed between these two groups with/without distractive stimulation, which indicates the DEGs are reliable and eligible for the following analysis.

### 2.2. GO and Pathway Enrichment Analyses

**Figure 4:**
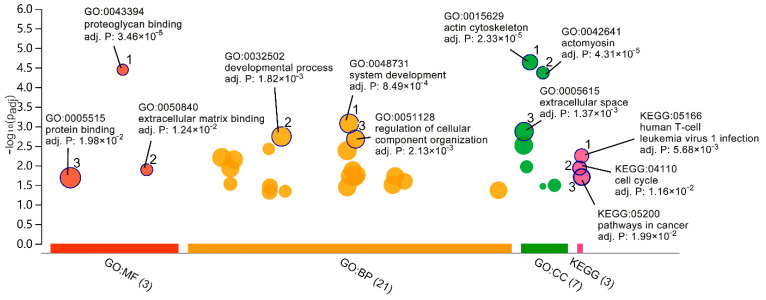
GO enrichment and KEGG pathway enrichment analysis. In the vertical direction, the higher the bubbles, the more significantly enriched. In the horizontal direction, terms from the same GO subtree are located closer to each other. Bubbles’ size stands for term size (gene quantity contained). The X-axis represents the group of functional terms and coloured by data sources, and the Y-axis lays out the adjusted *p*-value on the negative log10 scale.

The GO enrichment and KEGG pathway enrichment analyses were conducted to identify the biological function of DEGs. Figure 4 demonstrated enriched GO terms and KEGG pathways. In GO terms, system development, developmental process, and regulation of cellular component organisation were the most significant enrichments in the biological process. The actin cytoskeleton, actomyosin, and extracellular space were the most significant enrichment in the cellular component. Proteoglycan binding, extracellular matrix binding, and protein binding were the most significant enrichment in molecular function. In the KEGG pathway enrichment analysis, human T-cell leukaemia virus 1 infection, cell cycle, and pathways in cancer were remarkably related to the response of hBMSCs to distraction. A list of the top 5 enriched GO terms and KEGG pathways are shown in Table 1.

### 2.3. PPI Network Construction

**Figure 5:**
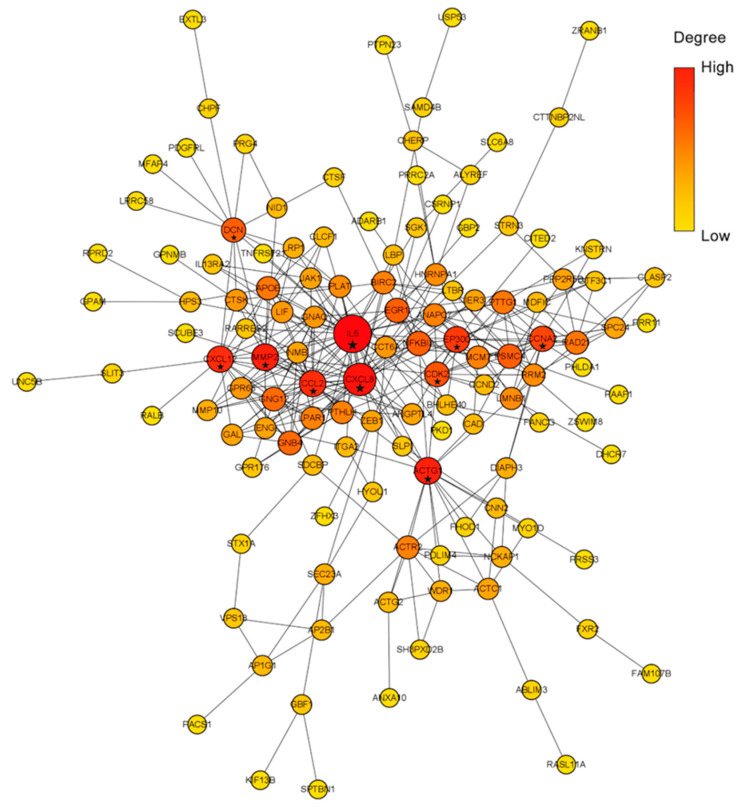
PPI network of all DEGs. Red indicates high connection degree, while yellow represents low. Node size also indicates the connection degree: the higher the degree is, the larger the node size. Nodes with star labels represent hub genes analysed by the cytoHubba.

The PPI network of all DEGs (Figure 5) constructed by the STRING database includes 126 nodes and 323 edges. In these *DEGs*, *IL6*, *CXCL8*, *MMP2*, *ACTG1*, *CCL2*, *CXCL12*, *EP300*, *CCNA2*, *CDK2*, and *DCN* were screened as ‘PPI hub genes’ according to the connection degree (Table 2). *IL6* displayed with the highest degree (degree = 38), followed by *CXCL8* (degree = 28). The deletion of *IL6* and *CXCL8* will remarkably loosethe structure of the PPI network and reduce the interaction between proteins. Therefore, *IL6* and *CXCL8* are the core nodes of PPI, suggesting that *IL6* and *CXCL8* play an important role in the response of hBMSCs to distraction.

### 2.4. Gene Set Enrichment Analysis

**Figure 6:**
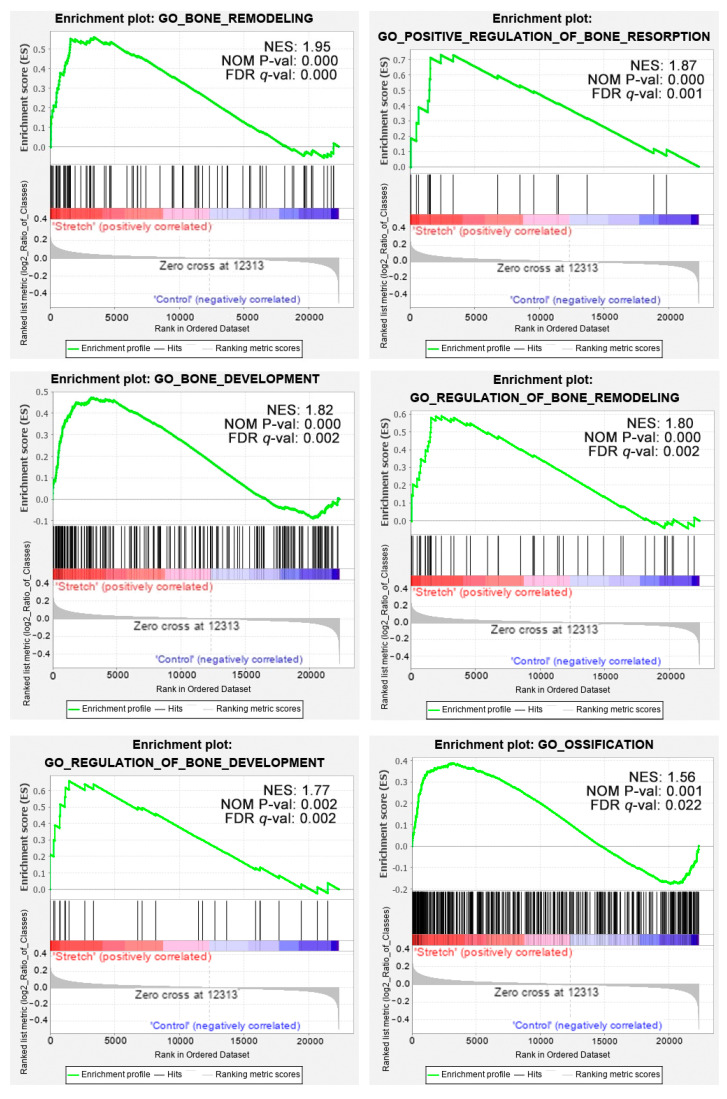
Enrichment plots from GSEA. GO terms of bone remodelling, positive regulation of bone resorption, bone development, regulation of bone remodelling, regulation of bone development, and ossification are significantly enriched in the distraction group.

Of the 19 osteogenesis-related gene sets, 5 were filtered out according to the exclusion criterion. The remaining 14 gene sets were utilised for GSEA. Among them, 13 gene sets were upregulated in the distraction group. Six gene sets were significantly enriched in the distraction group at the cut-off criterion |NES| >1, nominal *p* < 0.01, and FDR *q*-value < 0.25 (Figure 6).

### 2.5. Venn Diagram of Osteogenic Hub Genes

**Figure 7:**
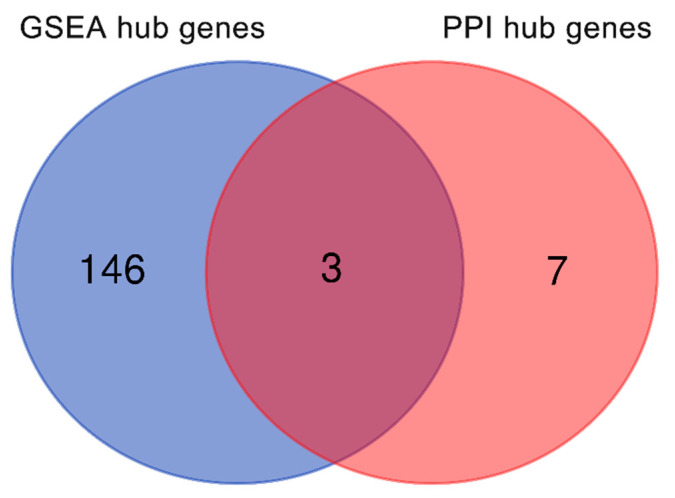
Venn diagram of hub genes. Blue represents GSEA hub genes, while red represents PPI hub genes. There were three genes in common between these two groups.

**Figure 8:**
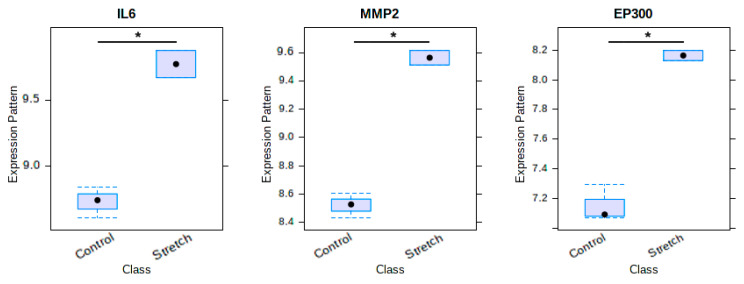
Expression patterns of identified genes associated with distraction osteogenesis between distraction group and control group. * *p* < 0.05.

There were 146 nonredundant ‘GSEA hub genes’ identified from the leading-edge subsets of the above six significantly enriched gene sets. These genes contributed the majority of enrichment signal so were the core of gene sets. Subsequently, we used the Venn diagram to analyse the ‘real’ hub genes associated with distraction osteogenesis between ‘GSEA hub genes’ and ‘PPI hub genes’. Finally, three overlapping genes, including *IL6*, *MMP2*, and *EP300*, were identified as the ‘real’ hub genes (Figure 7). All of these three genes showed a significantly upregulated expression level in the distraction group, compared with the control group (Figure 8).

## 3. Discussion

DO procedure has been widely accepted as a method of bone reconstruction by the orthopaedic community [3]. However, DO also encounters some knotty shortcomings. For example, bulky Ilizarov apparatus leads to physical stress due to the inconvenience of sleeping and personal hygiene, negatively impacting patients’ mental health. A long treatment period increases the risk of pin tract infections and hospitalisation expenses [2]. Reconstruction medicine is searching for novel methods that optimise and shorten the regenerative process. Increasing evidence has shown that locally delivered undifferentiated BMSCs have a positive effect on DO bone formation [5,9]. The mechanical stimulation of DO guides the fate of MSCs. Fang et al. indicated that cyclic stretch inhibited adipogenesis but facilitated osteogenesis of human adipose-derived MSCs, and tissue-regeneration-related cytokines were upregulated in the stretch group. Furthermore, PI3K/AKT and MAPK signalling pathways were activated by the cyclic stretch [16]. Although it has been reported that the cell fate is significantly influenced by the transduction pattern of external mechanical signals into the intracellular biological signals [17,18], the underlying biomolecular mechanisms for each type of mechanical stimuli remain to be elucidated. Therefore, it is of great significance to explore the mechanism of distraction-induced osteogenetic differentiation of MSCs. Bioinformatics methods have been widely applied to finding genetic changes in diseases, which is a reliable means of developing targeted therapy strategies.

Our bioinformatics analysis showed that 220 DEGs were identified between the distraction group and the control group. Compared with the control group, the enriched biological process GO terms in the distraction group were system development, developmental process, and regulation of the cellular component organisation. The proliferation-related pathway, including cell cycle and pathway in cancer, were enriched in KEGG pathway enrichment analysis. These findings are consistent with previous studies. The integrins on cytomembrane and their corresponding ligands on ECM transduce deformation caused by distractive or contractive forces into cells [19,20]. The direct alteration of the structural arrangement of the matrix mediates the local concentration and gradient of matrix-bound growth factors and adhesion sites, therefore affecting the proliferation and development of cells [21]. Ransom et al. [22] reported that DO upregulated core transcription factors (RUNX and DLX) that drive skeletal development, and the mechanotransducer focal adhesion kinase (FAK) transduced mechanical signals at integrin-mediated cell–matrix contacts into the nucleus, influencing proliferation, differentiation, and more.

Our bioinformatics analysis demonstrated that 10 genes, namely, *IL6*, *CXCL8*, *MMP2*, *ACTG1*, *CCL2*, *CXCL12*, *EP300*, *CCNA2*, *CDK2*, and *DCN*, were remarkably expressed in the distraction group. These genes were identified as closely related to the response to distractive mechanical stimulation of BMSCs. The following GSEA verified that 13 of 14 bone-formation-related gene sets were upregulated in the distraction group. Among them, six gene sets were significantly enriched in the distraction group. Three genes, namely, *IL6*, *MMP2*, and *EP300*, were overlapped between a group of 146 core enrichment genes from these six gene sets and the above 10 ‘PPI hub genes’. Therefore, we speculate that *IL6*, *MMP2*, and *EP300* are highly correlated with distraction-induced osteogenic differentiation of hBMSCs.

IL6 is one of the important pro-inflammatory cytokines involving in inflammation, immunoregulation, haematopoiesis, and tumourigenesis. During the early phase of fracture healing or the DO latency period, the upregulation of inflammatory genes due to the body’s own inflammatory response inevitably interferes with studying whether distraction could independently lead to the upregulation of *IL6* [23]. This issue was well avoided in our study because hBMSCs were the only research subject, and no extracellular inflammatory microenvironment was involved. Our results showed that distraction was independently responsible for the upregulation of *IL6*, and probably facilitated osteogenic differentiation. This concurs with the previous report by Cho et al. [24]. Their results indicated that both *IL1* and *IL6* were upregulated immediately after corticotomy but then fell to baseline levels rapidly during the postoperative period, whereas *IL6* alone was re-upregulated during the DO. Another study showed that *IL6* was induced within 24 h of distraction but not *IL1* [25]. These findings all suggest that *IL6* is especially sensitive to distractive stimuli. The potential mechanism of *IL6* influencing osteogenesis is that there may exist molecular crosstalk between the immune system and osteogenesis. Recent evidence showed that during the induced osteogenic differentiation of human adipose-derived MSCs, some Toll-like receptor agonists were capable of upregulating *IL6* expression. Importantly, *IL6* then appeared to induce the phosphorylation of STAT3 and subsequently activated the transcription of osterix, which is a vital transcriptional factor for osteogenic differentiation [26]. The IL6/STAT3 signalling may be of great significance in the *IL6*-mediated osteogenesis.

The migration of hBMSCs is one of the most important processes during the response to mechanical stimuli. The successful bone repair relies on MSCs migrating to bone formation areas [27]. MMP2 belongs to the MMP family of zinc-dependent proteolytic enzymes which was reported in participating in MSCs degrading surrounding ECM [28] and migrating to healing site [29]. MMP2 is a type of gelatinases and has high activity against gelatine, which facilitates the remodelling of ECM molecules. Yang et al. [30] indicated that distraction-induced the phosphorylation of p38, which then upregulated *MMP2* expression to degrade the ECM and promote migration. Similarly, other data supported that MMP2 played a key role in the angiogenesis, proliferation, and migration of MSCs [31]. Additionally, the balance between MMPs and their inhibitors, tissue-specific inhibitors of metalloproteases (TIMPs), is essential for osteogenic differentiation of MSCs in mechanical stimulation, and other members of the MMP family also participate in the osteogenesis [32]. Our study showed that *MMP2* was significantly upregulated by distraction and identified as a ‘real’ hub gene that may involve in distraction-induced osteogenic differentiation. Based on previous reports and our own findings, we speculate that for the specific distraction force, MMP2 may contribute more than other MMP family members. The enhanced migration ability of hBMSCs via p38/MMP2 signalling may be one of the multiple impacts of DO. The role of *MMP2* deserves further investigation.

Studies examined that mechanical unloading induced by simulated microgravity significantly downregulated *EP300* via the mechanosensitive microRNAs *miR-132-3p* in osteoblasts, which, in turn, led to inhibition of the activity and acetylation of RUNX2, a key regulator of osteoblasts differentiation [33,34]. The suppression of *miR-132-3p* resulted in the upregulation of *EP300* and led to enhanced osteogenesis [33]. A recent report further investigated the function of *EP300* in the osteogenic differentiation of mice BMSCs. Similar to previous studies on osteoblasts, the results indicated that the silence of *miR-132-3p* (target gene *EP300*) could effectively overcome the negative impacts of mechanical unloading on the osteogenic differentiation of BMSCs in vitro, and the bone quality was enhanced [35]. *EP300* was found one of the three ‘real’ hub genes in our study. These findings suggest that *EP300* also likely functioned as a key target for distraction-induced osteogenic differentiation of hBMSCs. To date, there are no data for how *miR-132-3p* and *EP300* were regulated by distraction in MSCs, which is worthy of being investigated in the future.

Although this study performed a comprehensive bioinformatics analysis, there were some shortcomings in this study. First, our study merely discussed the influence of short-term distraction on hBMSCs. The long-term effect of distraction remains unknown. Second, we utilised a reverse verification method in GSEA, which was verifying the enrichment of a self-defined gene sets collection. The results rely on the algorithm. The ultimate fate of hBMSCs in the original experiment has not been elucidated. Nevertheless, there were still some outlooks and values in our analysis. Third, this study lacks further validation. In vitro and in vivo experiments will be conducted in a future investigation.

## 4. Materials and Methods

### 4.1. Microarray Data Information

**Figure 9:**
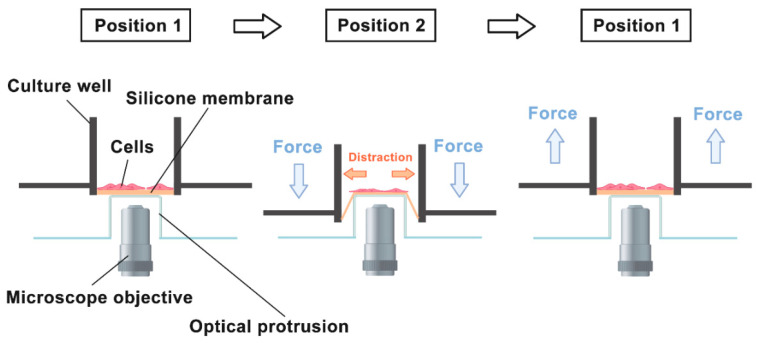
The schematic of self-designed distraction device. It is developed based on an inverted microscope for the purpose of real-time observation. Being mounted over the objective is an optical protrusion, on the top of that is a silicone membrane, holding hBMSCs in a culture well. There was cyclic force pressing (to position 2) or lifting (to position 1) the culture well sinusoidally, which made the silicone membrane stretched on the optical protrusion. The distraction was then transducted from the membrane into the intracellular parts of hBMSCs.

The gene expression profiles of E-MEXP-3124 were downloaded from a public functional genomics data repository known as the ArrayExpress database (https://www.ebi.ac.uk/arrayexpress/, accessed on 27 June 2020) [15] with the platform GPL6884 Illumina HumanWG-6 v3.0 expression beadchip (Illumina, San Diego, CA, USA). The datasets consist of 10 samples of mechanically stretched (and with or without adding Tubacin) hBMSCs (Cat. No. PT-2501, Cambrex BioScience, Rutherford, NJ, USA), of which cell source information was initially provided by the commercial company and can be found in the Appendix A Table A1. The original author verified hBMSCs’ pluripotency, before further experiments, by performing adipogenic and osteogenic differentiation tests on hBMSCs. Both of the Oil Red O and Alizarin Red S staining were positive Among these 10 hBMSCs samples, for the purpose of reducing interferences, only the samples without adding Tubacin were selected for analysis, including three samples of negative control and two samples subjected to 5% cyclic sinusoidal distraction at 0.25 Hz for 6 h. The distraction was applied using a self-designed device, which is schematised in Figure 9, as described by the original author. Another sample from the distraction group was disposed of due to being significantly inaccurate. Distraction experiments were carried out in a humidified incubator with 5% CO_2_ at 37 °C. Experiments without distraction were treated identically but not exposed to mechanical stress.

### 4.2. Identification of DEGs

Processed and quantile normalised plain text files were downloaded. The upregulated and downregulated DEGs between control groups and distraction groups were identified by the Limma method on the NetworkAnalyst 3.0 (https://www.networkanalyst.ca, accessed on 28 June 2020), which is a visual analytics platform for comprehensive gene expression profiling and meta-analysis [36]. Briefly, the process of DEG identification followed the instructions shown in each step. The *p*-value was corrected using the Benjamini–Hochberg test. Finally, the cut-off criterion of DEGs was set as log2 fold change |log2FC| > 1.0 and adjusted *p* < 0.05. A table containing identified DEGs was then generated and downloaded for further analysis.

### 4.3. GO and Pathway Enrichment Analyses

The identified DEGs were sorted out from the initial NetworkAnalyst table and copied to a new one (named DEGs table), which was then uploaded to the g:Profiler (http://biit.cs.ut.ee/gprofiler/, accessed on 1 July 2020), a public web server for characterising and manipulating gene lists resulting from mining high-throughput genomic data [37]. In this study, GO enrichment and KEGG pathway enrichment analysis of DEGs were performed via the g:GOSt on g:Profiler. The tailor-made g:SCS algorithm [37] and *p* < 0.05 were set as cut-off criteria. GO analysis comprises biological processes (BP), cellular component (CC), and molecular function (MF).

### 4.4. PPI Network Construction

In order to understand the mechanism to study the response of hBMSCs to distraction, and between proteins encoded by DEGs and different proteins, the STRING (https://string-db.org/, accessed on 5 July 2020) database [38] was utilised to recover the predicted associations between proteins encoded by DEGs and other proteins. The DEGs table was uploaded to the STRING to generate an interaction map of proteins coded by DEGs. A confidence score of >0.4 was defined as significant. The results of the interaction data were then downloaded and imported into the Cytoscape software (version 3.8.0, Cytoscape Consortium, San Diego, CA, USA) to visualise a PPI network. Degree distribution was figured by counting the number of connections between different proteins of the network. The plug-in cytoHubba was utilised to screen the top 10 hub genes ranked by degree.

### 4.5. Gene Set Enrichment Analysis

GSEA (https://www.gsea-msigdb.org/gsea/index.jsp, accessed on 12 July 2020) was performed to identify genes associated with osteogenesis. A self-defined osteogenesis-related collection was firstly created for this purpose. After screening, 19 annotated GO BP gene sets (C5 collection, full list is shown in Appendix B Table A2) in Molecular Signatures Database (MsigDB, version 7.1, https://www.gsea-msigdb.org/gsea/msigdb/index.jsp, accessed on 12 July 2020) were chosen and added into the self-defined collection as reference gene sets. The collection was then imported into GSEA software (version 4.0.3, Broad Institute, MA, USA) for further analysis. Gene sets that size smaller than 15 genes or larger than 500 genes were excluded prior to running analysis. The Signal2Noise method was selected for ranking genes. Gene set permutations were performed 1,000 times for each analysis to identify significantly different GO terms. The normalised enrichment score (NES), nominal *p*-value, and false discovery rate (FDR) *q*-value indicated the importance of the association between gene sets and GO terms. |NES| > 1, nominal *p* < 0.01, and FDR *q*-value < 0.25 were considered as statistically significant.

### 4.6. Venn Diagram of Osteogenic Hub Genes

A Venn diagram was plotted using the online Venn diagram web tool from the Bioinformation & Evolutionary Genomics (http://bioinformatics.psb.ugent.be/webtools/Venn/, accessed on 17 July 2020) to identify the ‘real’ hub genes associated with distraction-regulated osteogenic differentiation. The core enriched genes in the leading-edge subsets were referred to as ‘GSEA hub genes’, while the top 10 hub genes from PPI were referred to as ‘PPI hub genes’. The overlapping genes between these two groups were ‘real’ hub genes.

### 4.7. Statistical Analysis

The statistical package SPSS Statistics 22.0 (IBM, Chicago, IL, USA) was used for statistical tests. The normality was checked using the Shapiro–Wilk test. An independent-samples *t*-test was conducted to investigate the expression levels of ‘real’ hub genes. The significance value was taken as *p* < 0.05 in all statistical analyses. A full list of software and websites used in this paper can be found in Appendix C Table A3.

## 5. Conclusions

Our study analysed the gene expression profiles between distraction-induced and controlled hBMSCs. It provided new insights into key genes of osteogenic differentiation of hBMSCs during DO, and it could be used as new evidence and ideas for developing novel targeted therapy strategies to improve the therapeutic effects of DO. Three genes *IL6*, *MMP2*, and *EP300*, were identified as hub genes for distraction-induced osteogenesis of hBMSCs. These genes are more dominant in the response of hBMSCs to DO and are promising candidates for targeted therapies.

## Figures and Tables

**Table 1:** List of top 5 significantly enriched GO terms and KEGG pathways of DEGs.

Category	GO ID	Description	Gene Count	Adjusted *p*-Value
BP	GO:0048731	system development	83	8.49 × 10^−4^
BP	GO:0032502	developmental process	100	1.82 × 10^−3^
BP	GO:0051128	regulation of cellular component organisation	51	2.13 × 10^−3^
BP	GO:0022617	extracellular matrix disassembly	8	3.78 × 10^−3^
BP	GO:0048518	positive regulation of biological process	96	4.18 × 10^−3^
CC	GO:0015629	actin cytoskeleton	21	2.33 × 10^−5^
CC	GO:0042641	actomyosin mesenchymal transition	9	4.31 × 10^−5^
CC	GO:0005615	extracellular space	62	1.37 × 10^−3^
CC	GO:0005576	extracellular region	73	3.08 × 10^−3^
CC	GO:0005884	actin filament	8	1.08 × 10^−2^
MF	GO:0043394	proteoglycan binding	7	3.46 × 10^−5^
MF	GO:0050840	extracellular matrix binding	6	1.24 × 10^−2^
MF	GO:0005515	protein binding	156	1.98 × 10^−2^
KEGG	KEGG:05166	human T-cell leukaemia virus 1 infection	11	5.68 × 10^−3^
KEGG	KEGG:04110	cell cycle	8	1.16 × 10^−2^
KEGG	KEGG:05200	pathways in cancer	17	1.99 × 10^−2^

**Table 2:** The top 10 hub genes.

Rank	Gene Symbol	Degree
1	IL6	38
2	CXCL8	28
3	MMP2	17
3	ACTG1	17
5	CCL2	16
5	CXCL12	16
7	EP300	15
8	CCNA2	14
9	CDK2	13
10	DCN	12

## Data Availability

The gene expression profile E-MEXP-3124 can be downloaded from a public functional genomics data repository Array Express database (https://www.ebi.ac.uk/arrayexpress/, accessed on 27 June 2020).

## Appendix A

**Table A1:** Basic information about hBMSCs from the Cambrex BioScience.

Informations	Data
Donor Information	
Age	21 yo
Race	Black
Gender	Male
Cell Information	
Cell type	Human Bone Marrow Mesenchymal Stem Cell
Frozen Date	11 April 2006
Cell Passage	2
Number of cells	≥750,000 cells/0.5 mL–1,530,000 cells in total
Vitality-Trypan Blue	≥75%
Virus Tests	
HIV	Negative
HBV	Negative
HCV	Negative
Adipogenic Analysis	
Oil Red O Method	Positive
Chondrogenic Analysis	
Proteoglycan–Saffron Staining	Positive
Type II Collagen (on 14th and 21st days)	Positive
Osteogenic Analysis	
Calcium Deposition	Positive
Markers	
CD105, CD166, CD29, CD44	90% Positive
CD14, CD34, CD45	<5% Positive

## Appendix B

**Table A2:** Full list of self-defined osteogenesis-related gene sets collection.

Collection	Gene Set	Size
C5: GO BP	GO_DIRECT_OSSIFICATION	6
C5: GO BP	GO_BONE_REMODELING	91
C5: GO BP	GO_BONE_MINERALIZATION	112
C5: GO BP	GO_BONE_MATURATION	22
C5: GO BP	GO_BONE_GROWTH	47
C5: GO BP	GO_BONE_DEVELOPMENT	219
C5: GO BP	GO_OSSIFICATION	396
C5: GO BP	GO_OSSIFICATION_INVOLVED_IN_BONE_REMODELING	5
C5: GO BP	GO_POSITIVE_REGULATION_OF_BONE_DEVELOPMENT	9
C5: GO BP	GO_POSITIVE_REGULATION_OF_BONE_MINERALIZATION	39
C5: GO BP	GO_POSITIVE_REGULATION_OF_BONE_RESORPTION	19
C5: GO BP	GO_POSITIVE_REGULATION_OF_CELL_PROLIFERATION_IN_BONE_MARROW	8
C5: GO BP	GO_POSITIVE_REGULATION_OF_OSSIFICATION	88
C5: GO BP	GO_REGULATION_OF_BONE_DEVELOPMENT	24
C5: GO BP	GO_REGULATION_OF_BONE_MINERALIZATION	74
C5: GO BP	GO_REGULATION_OF_BONE_MINERALIZATION_INVOLVED_IN_BONE_MATURATION	5
C5: GO BP	GO_REGULATION_OF_BONE_REMODELING	48
C5: GO BP	GO_REGULATION_OF_OSSIFICATION	200
C5: GO BP	GO_REPLACEMENT_OSSIFICATION	28

## Appendix C

**Table A3:** Software and websites used in this paper.

Software/Website	Website Address
ArrayExpress database	https://www.ebi.ac.uk/arrayexpress/ (accessed on 27 June 2020)
NetworkAnalyst 3.0	https://www.networkanalyst.ca (accessed on 28 June 2020)
g:Profiler	http://biit.cs.ut.ee/gprofiler/ (accessed on 1 July 2020)
STRING database	https://string-db.org (accessed on 5 July 2020)
Cytoscape software (version 3.8.0)	https://cytoscape.org (accessed on 5 July 2020)
Gene set enrichment analysis software (version 4.0.3)	https://www.gsea-msigdb.org/gsea/downloads.jsp (accessed on 12 July 2020)
Molecular Signatures Database	https://www.gsea-msigdb.org/gsea/msigdb/index.jsp (accessed on 12 July 2020)
Venn diagram	http://bioinformatics.psb.ugent.be/webtools/Venn/ (accessed on 17 July 2020)
SPSS Statistics 22.0	https://www.ibm.com/support/pages/downloading-ibm-spss-statistics-22 (accessed on 23 July 2020)
